# Tobacco Interventions and Anaesthesia- A Review

**Published:** 2009-10

**Authors:** Usha Saha

**Affiliations:** Professor, Dept of Anaesthesiology, Lady Hardinge Medical College, Smt. Sucheta Kriplani & Kalawati Saran Childrens Hospital, New Delhi, 110001

**Keywords:** Tobacco, Smoking, Passive smoking, Second hand smoke, Health effects, Diseases, Lung cancer, Carcinogenesis, COPD, Anaesthetic considerations, Preoperative advice, Interventions

## Abstract

**Summary:**

Tobacco use is the leading preventable agent of death in the world. It is manufactured on a large scale in India and has a huge international market also. Death toll from tobacco use is on the rise. Use of tobacco is also increasing esp. in developing countries, in teenagers & in women, despite government, WHO and intervention by other statutory bodies. Prolonged use of tobacco or its products, as smoke or chew, endows significant risk of developing various diseases. With advances in surgical and anaethesia techniques & prolonged life expectancy, anaesthetist will be faced with management of these patients. Tobacco consumption affects every major organ system of the body; esp. lung, heart and blood vessels. Perioperative smoking cessation can significantly reduce the risk of postoperative complications & duration of hospital stay. Anaesthetist can play an important role in motivating these patients to quit smoking preoperatively by providing brief counselling and nicotine replacement therapy in reluctant quitters. More of concern is the effect of passive smoking (second & third hand smoke) on non smokers.

This is a review of tobacco & its products, their health consequences, diseases caused, anaesthetic considerations & their role in helping these patients quit smoking Preventing nicotine addiction and improving smoking cessation strategies should be the priority and despite these being only partially successful, strong measures at all levels should be continued & enforced.

## Introduction

Tobacco use is the leading preventable agent of death in the world. It is responsible for more than five million deaths each year & the death toll from tobacco is expected to climb to > eight million people per year within next 25 years. It is estimated that eventually 50% of all smokers will be killed by direct or indirect effects of tobacco. As in 2002, some 1.22 billion people smoked. It was predicted that by 2010, 1.45 billion people will smoke and 1.5 to 1.9 billion by 2025^1^.

The most popular type of substance that is smoked is **tobacco**. There are many different tobacco cultivars, which are made into a wide variety of mixtures and brands.

## History and Consumption

The history of smoking dates to 5000 BC. Early smoking evolved in association with religious ceremonies for purpose of spiritual enlightenment. The practice quickly spread from Europe & America to rest of the world^2^,^3^.

Perception surrounding smoking varied from being holy and sinful, sophisticated and vulgar, a panacea and a deadly health hazard^4^. Only recently smoking has come to be viewed in a complete negative light. Studies have proven that smoking is among the leading causes of many diseases such as lung cancer, heart attacks, etc^5^.

**Smoking** is the commonest method of consuming **tobacco**, and tobacco is the most common substance smoked, less common drugs being **cannabis** and **opium**.

**Smoking (dhumrapana** “**drinking smoke**” coined in 1700s) is a practice where tobacco is burned and smoke tasted, inhaled or actually drunk.

Tobacco **‘Brown gold’,** is an agro based product processed from fresh leaves of plants in genus **“Nicotiana’**. Of the several species; ***Nicotiana tabacum*** is commonly grown, but ***Nicotiana rustica*** also contains high concentrations of nicotine. The leaves are harvested, cured (slow oxidation and degradation of carotenoids in tobacco leaf)^6^, is treated, mixed with additives and then **pyrolyzed**. Tobacco is combined with upto 599 additives to enhance the addictive potency, improve the effect & make it more palatable. The resulting vapors are inhaled and active substances absorbed through lung

Tobacco has **Nicotine (2-5%,** +/- **0.23%)**, **Sugars** (mainly reduced) (8-25%, +/- 1.8%) and **Moisture** (10-14% +/- 0.3%).

The word **‘Nicotine’** is derived from Frenchman **Jean Nicot** who introduced tobacco to France in 1560 It is consumed in two forms, as -

1. **Smoke**
2. **Smoke less**

### Smokes

Tobacco for smoking is available as **Beedi**^7^ (higher levels of CO, nicotine, and tar), **Cigar**, **Cigarettes, Electronic cigarette** (provides nicotine vapor from nicotine solution), **Hookah** 8 (operates by water filtration and indirect heat), **Kreteks** (cigarettes introduced in Java) and **Pipe. Vaporizer** is used to sublimate the active ingredient in partial vacuum, rather than burning, with less production of irritating, toxic, carcinogenic by-products. Each cigarette can cause much damage (Table 1)

**Table 1:** Facts about one cigarette

On average, each cigarette shortens a smoker's life by around 11 minutes. A single cigarette can reduce the blood supply to skin for over an hour. Cigarettes contain more than 4000 chemical compounds, at least 400 are toxic. When one inhales, a cigarette burns at 700°C at the tip and at 60°C in the core. The British Medical Association estimates that up to120,000 men have ED because of smoking.

## Non smoking exposures

Even non smokers are not exempt from the adverse health effects of smoking. Because of its negative implications; this form of consumption has played a key role in regulation of tobacco products. **Smokeless tobacco** also contains nicotine. **Passive smoking** is involuntary consumption of tobacco smoke & falls into 3 categories^9^-

1. **Second-hand smoke (SHS)**
2. **Environmental tobacco smoke** (ETS)
3. **Third-hand smoke-**smoke that remains after burning end has been extinguished
4. **Snuff-**fine-ground smokeless tobacco product, introduced into nostrils
5. **Chewing tobacco-** put inside the mouth

## Health effects-

- It is highly **addictive.** Within 30 mins, nicotine equivalent to 4 cigarettes is ingested, and easy to become dependent upon
- Contains > **25 carcinogens,** with 50% higher risk of Oropharyngeal cancer
- Leukoplakia-from continued irritation of gums, tongue and oral mucosa
- Increased risk of CVS diseases
- Permanent discolouration of teeth, halitosis and gum disease

**Physiology** Acetylcholine and Nicotine are chemically similar. Nicotine triggers cholinergic receptors, releasing adrenaline & nor adrenaline from adrenals, besides dopamine and endorphins. This gives a pleasurable sensation, referred to as a “high” ranging between mild stimulus caused by nicotine to intense euphoria caused by heroin, cocaine and amphetamines Common result of smoking is the characteristic facial change known as smoker's face.

When tobacco is smoked, most of the nicotine is pyrolyzed, but remaining is sufficient to cause somatic and psychological dependency. **Harmane (MAO inhibitor**) formed from acetaldehyde in tobacco smoke, has a role in nicotine addiction Not all drugs can be smoked, e.g. sulphate derivative is most commonly inhaled through nose, while purer free base forms require skill in administering. Also, not all smoke will be inhaled.

Cigarettes contain more than 4000 chemical. At least 400 are toxic. On inhaling, a cigarette burns at 700°C at the tip and 60°C in the core. This burns tobacco by incomplete combustion, to various toxic substances.As a cigarette burns, the residues get concentrated towards the butt. The products most damaging are:

1. **tar**- a carcinogen.
2. **nicotine**- addictive and increases blood cholesterol, and
3. **carbon monoxide** (CO)- reduces O_2_ delivery to tissues

## Consequences of smoking^10^

- Causes cancers in most organs of the body, including kidney, cervix, and bone marrow, not previously linked to smoking.
- Components of the gas and particulate phases cause COPD. The damage caused is influenced by the number of cigarettes smoked, filtered or not and method of tobacco preparation
- Also causes cataracts and osteoporosis and increased risk for fractures.
- Poor general health. Adverse effects begin before birth and continue across the life span
- Reduced life expectancy by seven to eight years. The number of people < 70 yrs age, who die from smoking-related diseases exceeds the total deaths caused by breast cancer, AIDS, traffic accidents and drug addiction.

## Tobacco & cancer

In 18^th^ century, London physician Percival Pott made the first link between cancer and environmental agents when he noted a high incidence of scrotal cancer among chimney sweeps and hypothesized the cause being exposure to coals and tars^9^.

**Lung cancer** is a leading cause of **cancer** death. Worldwide > one million people die each year. Cigarette smoking is the major cause of **lung cancer**. The risk diminishes after smoking cessation for > 5 years, but relative risk is still more than of non-smokers. The carcinogenic mechanism of **tobacco** smoking is a complex process. The tar fraction of cigarette smoke includes both initiators and promoters of carcinogenesis, making it especially dangerous.

The **mainstream smoke** from the mouthpiece of a cigarette is an aerosol (10^10^ particles/mL). About 95% of smoke (vapor phase) is made of N_2_, NO, O_2_ & CO_2._

The particulate phase contains at least 3500 compounds, most carcinogens and free radicals. The major free radical species is quinone-hydroquinone complex “held in a tar matrix”. Free radical complex causes redox cycling, generating superoxides from O_2_ with formation of H_2_ O_2_, OH ions & DNA nicking. Nitricoxide acts synergistically with “tar” to cause DNA breakage.

Gas phase causes lipid peroxidation of blood plasma & formation of carbonyls (prevented by ascorbic acid). Ascorbic acid levels are lower in smokers. Daily consumption of > 200 mg of Vit C / day is required for serum ascorbate levels to be similar to those in nonsmokers.

There are about 55 carcinogens in cigarette smoke. (Table 2). Some of them are:-

1. **Benzo[*****a*****]pyrene (BaP)** (PAH)- is a potent **lung** carcinogen
2. The **tobacco**-specific ***N*****-nitrosamine** (NNK).
3. **1,3-Butadiene** and **Ethyl carbamate**
4. **Nickel, chromium, cadmium, and arsenic**
5. **Polonium-210**
6. **Hydrazine**
7. **Toxins** – Acrolein, Nitrogen oxides and Acetaldehyde
8. Weakly acidic compounds (**Tumor Promoters)** -Catechols, Pyrogallol, Decane, undecane, Pyrene, benzo[*e*]pyrene, and Fluoranthene

**Table 2:** List of Carcinogens

Agent	Cancer Type
**Benzo [a]-pyrene (Tobacco)**	Lung
**Alcohol**	Mouth, Pharynx, Larynx, Esophagus
**Dietary Fat**	Breast
**Asbestos**	Respiratory-tract, Pleural and Peritoneal Mesothelioma
**Fermented Foods**	Stomach
**Estrogens**	Endometrial, Ovarian, Breast
**UV Light**	Skin
**Gamma Radiation**	Leukaemia, Thyroid, Breast, Lung, Mouth, GIT, Bladder, Ovarian, Skin
**Aflatoxin**	Liver
**Soot, Coal (Chimney Sweep)**	Scrotal
**Nickel (Nickel Refining)**	Lung, Nasal
**Wood Dust (Woodworking)**	Nasal
**Cr(VI) (Leatherworking)**	Lung
**Mustard Gas**	Respiratory-tract, Lung
**2-napthylamine**	Bladder
**hepatitis B & C**	Liver

**Sidestream smoke,** released from the tip of a cigarette plus that which diffuses through cigarette paper, constitutes the major portion of ETS. Ratio of carcinogens in sidestream to mainstream smoke is >1, but dilution with air ensures that passive uptake will be far less than in a smoker.

**Measures of cigarette smoke uptake-**Various biochemical markers to measure cigarette smoke uptake used, are:-

1. **Exhaled CO, Carboxyhemoglobin and Thiocyanate**
2. **Urinary mutagenic metabolites** NNK metabolites NNAL and NNAL-Gluc. NNAL is a potent pulmonary carcinogen, while NNAL-Gluc is not. Ratio of the two is used to assess susceptibility to **lung cancer.** This ratio is very low in black than in white smokers, suggesting poor detoxification as a factor contributing to higher incidence of **lung cancer** in blacks
3. **Cotinine,** a nicotine metabolite, is the most specific and widely used

### Health hazards (Fig 1)

Smoking impairs physical fitness, general well being and endurance. Tobacco use affects every system of the body. Nicotine in cigarette smoke can disturb the functioning of the inner ear, sense of balance & dizziness. Most commonly affected organs are heart and lungs. Smoking is a major risk factor for heart attacks, strokes, COPD, cancer, fertility and pregnancy related problems, birth defects, blindness etc.

**Fig 1:**
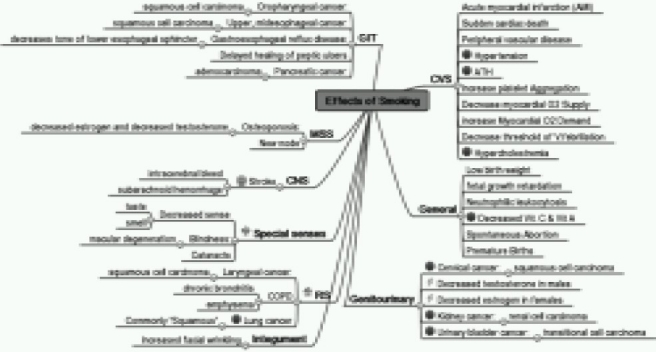
Health hazards of smoking Stephen Hecht JNCI J Natl Cancer Inst Volume 91, Number 14 1999

## Cardiovascular Effects

Tobacco in any form trebles the risk of cardiac disease. About 30% of all deaths from heart disease are due to smoking. Cardiovascular effects of smoking occur within minutes with rise in HR upto 30% in first 10 mins. This is short lived, but since most smoke cigarettes several times a day, these occur often, leading to long-term problems^11^,^12^.

## Atherosclerosis

Atherosclerosis is a normal aging process but smoking accelerates it. Depending on which blood vessels are involved, symptomatology varies.

- **Coronary vessels**: leading to IHD or MI. Smokers develop coronary thrombosis 10 years earlier than non-smokers, & 9 out of 10 patients who undergo CABG, are smokers.
- **Cerebral vessels:** leading to **collapse**, sub arachnoid hemorrhage (SAH), **stroke, paralysis, coma & death**.
- **Renal arteries-** leading to renal hypertension or renal failure
- **Peripheral arteries**- leading to peripheral vascular disease (Burgers disease, Raynaud's phenomenon), and **gangrene**.
- **Cardiomyopathy**-CO in smoke damages the heart muscle & increases myocardium's susceptibility to viral infections, cardiomyopathy and CCF.
- **Eye–** vision disturbances, blindness, optic neuropathy & macular degeneration

## Cancer

Lung cancer was a rarity before the invention of cigarettes. Smokers are 5-10 times as likely to develop lung cancer. Each year, > one million smokers die of lung cancer in USA, accounting for 25% of total smoking-related deaths^13^. 1 in 10 moderate smokers and 1 in 5 heavy smokers (>15 cigarettes/day) will die of lung cancer. About 85% of smokers with lung cancer die within 5 yrs & > half die within one year.

Smoking also causes cancer on lip, tongue, or other locations in the mouth, often requiring surgery, Cancer of nose, (rare, less fatal), Pancreas (80% die within a year), Oesophagus (cause of 80 to 90% of oesophageal cancers, always in combination with alcohol use), Bladder, & Cervix (probably due increased susceptibility to sexually transmitted virus)^14^.

## Respiratory system

Smoking impairs pulmonary functions by damaging the cilia, alveoli, and bronchioles & increasing irritability of the bronchial tree^15^. Smoking is the most common cause of COPD. It's estimated that 94% of 20-a-day smokers have some emphysema when examined post mortem, while > 90% non-smokers have none. It starts between the ages of 35 and 45 when lung function starts to decline anyway. In smokers, the rate of decline trebles with onset of symptoms.

## Management^16^,^17^

Of foremost importance is Cessation of Smoking

- Patient should avoid being around smokers and fume-laden polluted air
- Use a face mask or nose filter
- Those above 60 yrs age must receive Influenza & Pneumonia vaccination
- Maintaining hydration & healthy diet
- Deep breathing exercises (Belly breathing)
- Physical activity (exercise, walking and yoga)
- Controlled Oxygen therapy increases survival rates, alleviates symptoms, and improves quality of life in patients with severe COPD
- **Antibiotics**
- **Bronchodilators-** beta_2_ agonist and ipratropium ***(Combivent)***
- **Mucolytics**
- **Corticosteroids** –routine use is not advocated. Oral or inhaled are useful in acute exacerbation (shorten recovery time; improve FEV_1_ & oxygenation, decreased relapse and shorter length of hospital stay). Common corticosteroids are- Flovent, Solumedrol and Prednisone
- **Other medications–** NRT, antidepressants, Clonidine (in hypertensives, helps in withdrawal symptoms)
- **Management** of complications of COPD e.g., heart failure, pulmonary hypertension
- **Long-term oxygen therapy** if hypoxemia, pulmonary hypertension, respiratory failure are present
- **Newer Developments -**Antitrypsin replacement therapy-for inherited deficiency of a antitrypsin in emphysema
- **Pulmonary rehabilitation -** for end stage COPD. There are three types of surgical options **- Bullectomy, Lung Volume Reduction Surgery (LVRS)** and **Lung Transplantation.**

**Kidney -** Smoking causes and worsens renal damage in those with medical problems affecting kidneys, e.g. diabetes mellitus or hypertension. Urinary tract problems are more common among smokers, because tobacco components in urine irritate urinary tract, causing frequent micturation & nocturnal dysuria.

**Eye-**Heavy smokers are more likely to get cataract^18^. Exhaled

**Infections & immunity -** Smoking interferes with cell mediated immunity and makes smokers prone to gum infections and periodontal diseases^19^.

**Bone and muscle pain -** Smokers are more likely to have back ache, bone and muscle pain & injuries, partly due to compromised peripheral circulation.

**Smoking and Thyroid Disease -Cyanide,** in tobacco smoke, interferes with thyroid hormone production. Smoking triples the risk for developing thyroid disease and goitre. Women smokers with sub clinical hypothyroidism have a high risk for developing full-blown hypothyroidism.

## Reproductive system

Women are more likely to have menstrual irregularities, infertility problems, cramps & hot flashes during menopause. Smoking lowers estrogen levels and attain early menopause with increased risk of osteoporosis & fractures.

## Problems during pregnancy^20^

**Immediate problems -** Continuing to smoke during pregnancy carries risks for the foetus due to lower immunity, CO in maternal blood & hypoxemia

- Greater risk for ectopic, miscarriage, premature rupture of membranes, placenta previa, and abruptio
- Effect on foetus-increased risk for stillbirth, prematurity, and low birth weight babies
- Increased risk of babies born with cleft lip

**Long-term problems in children born to smoking mothers–**

- Increased risk of allergies
- Childhood hypertension, obesity, diabetes mellitus
- Poorer lung functions & chances of developing early asthma
- Increased risk of mental retardation, learning and behaviour problems

**Smoking & elderly-**Elderly face increased risk of fractures, cataracts, and COPD. Certain age-related conditions occur at higher rates and earlier in smokers e.g. Cataracts, Age-related macular degeneration (AMD), Gum disease, tooth loss, Wrinkles, Baldness, premature greying, Hearing loss, urinary & faecal incontinence.

**Non smoking dangers of tobacco -Fire deaths**- Smoking causes 6% of all fires, but it is the leading cause of deaths in fire accidents. Smoking-related fires are deadlier because they occur in homes, at night, when everyone is asleep^21^,^22^.

**Smoking and Surgical Recovery-S**moking increases the risk of perioperative cardiac & respiratory problems, delayed wound healing and prolonged hospital stay.

## Quitting^10^

The benefits of quitting begin within 20 minutes of the last cigarette.

- At 8 hrs–BP & PR decreases, CO level in blood drops, O_2_ levels & body temperature increase to normal.
- At 24 hours-Chance of heart attack decreases
- By 3 Months-PFT improve, & worst of nicotine withdrawal symptoms subside.
- By 9 Months-clinical improvement in respiratory symptoms occurs.
- By Year-there is 50% reduction in risk of CAD
- By 5 years-CVA & CAD risk is reduced to same as non smokers
- By 10 years-50% decrease in risk of lung & other cancers
- By 15 years-risk of CAD & death becomes similar to non smokers

## Anaesthetic interventions^23^

Up to now, primary care physicians have been the focus of health care system efforts to address smoking. Cigarette smoking exacts an enormous toll in human suffering and economic costs. The risk of premature death and disability is dramatically reduced when smokers quit, even if they have already developed smoking-related disease or have smoked for decades. Specialists like anaesthesiologists were ignored**.** A Task Force of the ASA appointed in 2006, adapted the evidence-based US clinical practice guidelines for physicians into a strategy relevant and efficient for anaesthesiologists^24^.

## Why should anaesthesiologists bother to address tobacco use?

1. First reason - Doing something about a patient's smoking can improve short term clinical outcomes. Smoking is an important risk factor for perioperative cardiac, respiratory, and wound healing complications. Also, those who quit 3 weeks preoperatively have reduced risk of perioperative complications and shorter hospital stay. Even shorter period of abstinence has some benefits^25^,^26^
2. Second reason - Anaesthesiologists encounter, smokers at a unique moment. Patients facing surgery feel vulnerable and are eager to comply to reduce one's risk of surgical complications. Surgery under anaesthesia is a powerful motivation to change behavior. Preop counseling and use of quitting aids increases the proportion of quitters^25^.
3. Third reason - Smoking interventions in the hospital increase the odds that a smoker will quit longterm after discharge as long as there is continued support^27^.

The ASA's Smoking Cessation Initiative Task Force **has designed a simple three-step system (Ask-Advise-Refer or AAR) that tailors evidence-based smoking cessation strategies** for anaesthesiology practice. It is designed to be quick and efficient to implement^28^. Here, the physician-

A- Asks a patient about smoking status

A- Advises smokers to quit, and

R- Refers smokers to free national telephone quitline

## Anaesthetic management

As far as possible patient's condition should be optimized preoperatively and all procedures undertaken as elective, except for emergency or life saving procedures.

Choice of anaesthesia technique is dictated by the surgical procedure to be undertaken. Neuraxial or regional anaesthesia should be preferred wherever possible, keeping in mind the increased risk of complications following GA in smokers because of adverse respiratory (hyperirritable respiratory tree) & cardiovascular effects. Approach to management remains the same as for non smokers.

Irrespective of the anaesthesia technique to be used, a thorough detailed preoperative evaluation should be done with medical and drug history, history of allergies, details of tobacco use (amount, duration) and past experiences with anaesthesia. Alcohol increases the risk of anaesthesia in smokers, & this history should be established. History regarding each major organ system must be established (cardiac, CNS, respiratory, renal, hepatic and GIT).

A thorough general physical & systemic examination must be carried out to confirm the findings in history, or establish a new one.

Besides routine investigations, complete blood counts (TLC, DLC), LFT, KFT, X ray chest, pulmonary function tests, blood gas analysis, electrolytes, ECG, echocardiography and blood sugar (for diabetic control) must be done. Thyroid functions need to be done if suggested by the history, besides investigations pertaining to the surgical procedure.

## Preoperative advice –

- Fasting overnight or for at least 6 hours Preop
- Smoking cessation-advise fiber rich, lots of fruits, vegetables, plenty of water, milk, calcium & Vit D supplements, strengthening exercises, nicotine replacement therapy (NRT),Alternative therapies such as acupuncture and hypnosis
- Care for smoking related medical diseases
- Care for incidental medical problems
- Preop and post treatment PFT

## Intraoperative

Great care needs to be exercised to prevent-

- Respiratory complications such as laryngo or broncho spasm (hyperirritability)
- Hemodynamic disturbances
- Careful dosing of drugs esp. relaxants
- Care with narcotics, esp. morphine. Short acting drugs (fentanyl, remifentanil, sufentanyl) and NSAIDS should be preferred.
- Maintaining adequate hydration & output
- Care with ventilation- IPPV with small tidal volume & higher rate.
- Avoid PEEP, high FiO_2_, hyperventilation (CO_2_ wash-out)
- With neuraxial block-avoid over transfusion & precipitation of CCF

**Intra op monitoring** –non invasive monitoring is sufficient for most operations for HR, BP, temperature & respiration, FiO_2_, EtCO_2_, SpO_2_, intake output, arterial blood gas analysis, CVP

## Indications for invasive monitoring-

- ASA 3 or 4 patients
- Extensive general surgical operation
- Cardiac surgery
- Neurosurgical procedures
- Organ transplants
- Hepatic resections, etc

## Post op instructions -

- Monitoring to be continued for at least 6-8 hours
- Controlled O_2_ therapy
- No driving or use of machinery for at least 24 hours after GA
- No Alcohol or unprescribed drugs
- Motivate patient to continue to remain tobacco free

**Post operative side effects -** Smokers have more complications following anaesthesia. Some of these are:-

- Delayed recovery
- Respiratory infections
- Prolonged hospital stay
- Excessive drowsiness, dizziness and headache (from CO_2_ washout, hypertension)
- Nausea and vomiting
- Shivering –should be avoided (increases O_2_ consumption)

Most side effects wear off within 24 hours. Risk of complications depends on patient's age, sex, weight, current medical condition and use of alcohol or drugs

Changes in cigarettes to reduce yields of tar and nicotine have no benefits. Hence, measures to prevent smoking initiation need to be strong and enforced, especially among young adults.

Although current rates of intervention provided by anaesthesiologists and surgeons are low, there is considerable interest among these physicians in learning more about interventions. Given the relatively high prevalence of smoking in Japan and the potential for surgery to serve as a **‘teachable moment’** to promote abstinence from smoking, leadership by these specialists in the area of tobacco control could have a major impact on public health in Japan^29^,^30^.

There is insufficient evidence about long-term benefit to give firm support the use of interventions intended to help smokers reduce tobacco use. Some people who do not wish to quit can be helped to cut down the number of cigarettes smoked and reduce their CO levels by using NRT. Preventing nicotine addiction and improving smoking cessation strategies should be the priority, but despite these being only partially successful, strong measures at all levels should be continued & enforced.
